# Maternal Serotonin Reuptake Inhibitor Antidepressants Have Acute Effects on Fetal Heart Rate Variability in Late Gestation

**DOI:** 10.3389/fpsyt.2021.680177

**Published:** 2021-08-16

**Authors:** Kayleigh S. J. Campbell, Abby C. Collier, Michael A. Irvine, Ursula Brain, Dan W. Rurak, Tim F. Oberlander, Kenneth I. Lim

**Affiliations:** ^1^BC Children's Hospital Research Institute, Vancouver, BC, Canada; ^2^Department of Obstetrics and Gynaecology, University of British Columbia, Vancouver, BC, Canada; ^3^Faculty of Pharmaceutical Sciences, University of British Columbia, Vancouver, BC, Canada; ^4^Department of Pediatrics, University of British Columbia, Vancouver, BC, Canada

**Keywords:** serotonin reuptake inhibitor antidepressants, prenatal exposure, fetal heart rate variability, sex differences, antidepressant pharmacokinetics, maternal depressed mood, pregnancy, third-trimester

## Abstract

**Background:** Prenatal exposure to serotonin reuptake inhibitor (SRI) antidepressants increases risk for adverse neurodevelopmental outcomes, yet little is known about whether effects are present before birth. In relation to maternal SRI pharmacokinetics, this study investigated chronic and acute effects of prenatal SRI exposure on third-trimester fetal heart rate variability (HRV), while evaluating confounding effects of maternal depressed mood.

**Methods:** At 36-weeks' gestation, cardiotocograph measures of fetal HR and HRV were obtained from 148 pregnant women [four groups: SRI-Depressed (*n* = 31), SRI-Non-Depressed (*n* = 18), Depressed (unmedicated; *n* = 42), and Control (*n* = 57)] before, and ~5-h after, typical SRI dose. Maternal plasma drug concentrations were quantified at baseline (pre-dose) and four time-points post-dose. Mixed effects modeling investigated group differences between baseline/pre-dose and post-dose fetal HR outcomes. *Post hoc* analyses investigated sex differences and dose-dependent SRI effects.

**Results:** Maternal SRI plasma concentrations were lowest during the baseline/pre-dose fetal assessment (trough) and increased to a peak at the post-dose assessment; concentration-time curves varied widely between individuals. No group differences in fetal HR or HRV were observed at baseline/pre-dose; however, following maternal SRI dose, short-term HRV decreased in both SRI-exposed fetal groups. In the SRI-Depressed group, these post-dose decreases were displayed by male fetuses, but not females. Further, episodes of high HRV decreased post-dose relative to baseline, but only among SRI-Non-Depressed group fetuses. Higher maternal SRI doses also predicted a greater number of fetal HR decelerations. Fetuses exposed to unmedicated maternal depressed mood did not differ from Controls.

**Conclusions:** Prenatal SRI exposure had acute post-dose effects on fetal HRV in late gestation, which differed depending on maternal mood response to SRI pharmacotherapy. Importantly, fetal SRI effects were sex-specific among mothers with persistent depressive symptoms, as only male fetuses displayed acute HRV decreases. At trough (pre-dose), chronic fetal SRI effects were not identified; however, concurrent changes in maternal SRI plasma levels suggest that fetal drug exposure is inconsistent. Acute SRI-related changes in fetal HRV may reflect a pharmacologic mechanism, a transient impairment in autonomic functioning, or an early adaption to altered serotonergic signaling, which may differ between males and females. Replication is needed to determine significance with postnatal development.

## Introduction

Up to 20% of women experience depressed mood during pregnancy (1, 2), and nearly one half of these women are treated with a serotonin reuptake inhibitor (SRI) antidepressant (3). Since their introduction nearly 30 years ago, the decision to start, continue or discontinue SRI antidepressant treatment during pregnancy remains complex, as clinicians and women continue to weight risks of adverse outcomes against relapse (4, 5). Prenatal SRI exposure has been associated with increased risks for preterm birth, lower birth weight and neonatal behavioral disturbances (6), as well as altered stress-regulation, social-emotional behaviors and other neurodevelopmental outcomes from infancy-to-childhood (7–13). However, many of these long-term associations may be confounded by the underlying maternal psychiatric disorder (14). Antenatal maternal mood disturbances are, similarly, associated with altered neurobehavioral outcomes in infancy (15–17), stress-regulation in childhood (18) and a risk for later psychopathology, emotional, or behavioral disturbances (19). Whether the early origins of these outcomes are already evident before birth remains unclear. This study was undertaken to investigate the effect of prenatal exposure to SRIs on fetal heart rate (HR) and relationships to maternal antidepressant pharmacokinetics in late gestation, controlling for the effects of depressed mood.

Both maternal psychiatric distress and its treatment with SRI antidepressants are early exposures that may influence the *in utero* environment (20–22), possibly through the modulation of fetal, maternal, or placental serotonin (5-hydroxytryptamine; 5-HT) signaling (23, 24). In particular, SRIs act by inhibiting the reuptake of the extracellular 5-HT leading to an increased duration and magnitude of serotonergic activity on pre- and postsynaptic receptors. During development, 5-HT is present from early gestation (25) and has been identified as a key neurotrophic factor regulating the construction and plasticity of neuronal circuits within its own and non-serotonergic systems (26). Across the lifespan, 5-HT also has extensive roles in neuropsychological and other central, autonomic, and peripheral nervous system processes (27). As SRIs are lipophilic compounds with high placental permeability, it is conceivable that altered 5-HT signaling before birth may have broad neurodevelopmental and physiologic implications.

To date, only few studies have investigated whether outcomes of prenatal SRI exposure emerge before birth: during the period of drug exposure. Fetal SRI exposure has been associated with disrupted cardiovascular function (28–30) and increased fetal motor activity (28, 31–33); though, findings are not consistent, primarily due to variations in methodology, gestational age and the ability to account for maternal mood. In SRI exposed fetuses, Mulder et al. report increased motor activity in the second trimester and increased motor activity during quiet sleep state (i.e., stable fetal HR, low variability) in the third-trimester; however, SRI-treated mothers had comparable psychiatric symptoms to the unmedicated depressed group (28). Gustafsson et al. also observed increased motor activity in SRI-exposed fetuses, but only prior to 30-weeks' gestation and found no SRI-related effect on fetal HR, HR variability, or HR-movement coupling (31). Conversely, lower fetal HR variability at 36-weeks' gestation and reduced cerebral blood flow resistance was observed in SRI-exposed fetuses (30), as well as elevated pulmonary blood flow in SRI-exposed fetuses who experienced transient respiratory difficulties at birth (29). Critically, outcomes from previous fetal SRI studies remain confounded by maternal psychiatric symptoms. A case in point, altered fetal motor activity, HR and HR variability have also been associated with antenatal maternal depression (34–37) and anxiety [e.g., reviewed in (38)]. Thus, investigating fetal outcome related to prenatal SRI exposure requires appropriate control groups for maternal mood.

Fetal outcome may also be differentially sensitive to acute and chronic drug effects, whereby outcomes vary depending on the time of assessment relative to SRI exposure. In fetal sheep studies, acute and chronic SRI effects have been observed, with transient reductions in uterine blood flow and reduced fetal oxygenation status following acute SRI infusion (39), but a sustained decrease in low-voltage electrocortical fetal brain activity with prolonged SRI exposure (40). While it is presently unknown whether SRI exposure has distinct acute and chronic effects on human fetuses, a pharmacologic mechanism has been suggested (41, 42). SRI dose-relationships with fetal, obstetric, and neonatal outcomes have been reported (28, 43, 44), and there is high correspondence between maternal and fetal plasma drug concentration ratios in amniotic fluid (45) and cord blood (46, 47) that vary with SRI type. Importantly, fetal exposure to other psychoactive agents have produced differential acute outcomes, such as an acute suppressive effect of buprenorphine on fetal HR and movement (48) and decreased fetal HR variability following acute nicotine exposure (49). Together, these studies suggest that fetal HR may be sensitive in detecting differences between acute and chronic psychotropic drug exposures.

Fetal HR and its variability are prenatal markers of cardiovascular regulation and can be studied non-invasively using Doppler ultrasound-based technologies, such as cardiotocography. Fetal HR and HR variability are widely described as indies of early autonomic functioning (50, 51), and the coalescence of fetal HR patterns and accelerations with motor activity around 32-weeks' gestation is viewed as organized neurobehavior (52–54). As the fetus matures, the well-characterized decrease in fetal HR and increase in HR variability (52, 55–58) are thought to reflect increasing sympathetic responsiveness and an emerging influence of parasympathetic (i.e., vagal) modulation (51). Fetal HR variability has been described as a psychophysiological construct with behavioral trait-like correspondence (50), reflecting an individual's emerging capacities for adaptive flexibility and interaction with environment, serving to prime the fetus for extrauterine life (59). Fetal cardiac patterning demonstrates developmental stability into the postnatal period, as it's highly correlated with neonatal and infant HR (60) and predicts temperament and neurodevelopmental outcomes in infancy (61–63), as well as behavioral regulation in childhood (64).

The present study was undertaken to investigate acute and chronic effects of prenatal SRI antidepressant exposure on fetal HR and HR variability in late gestation, while evaluating the concurrent effects of prenatal maternal depressed mood. Chronic effects of SRI exposure were determined by comparing fetal outcomes at a baseline period prior to typical morning oral SRI dose (i.e., pre-dose; at pharmacologic trough). Acute SRI-exposure effects were determined at peak drug levels (~4–5 h post-dose). Maternal SRI plasma drug concentrations across five time-points were used to characterize pharmacokinetics and assess drug level changes relative to periods of chronic and acute SRI exposure. To distinguish SRI-related effects from prenatal maternal depressed mood, we compared fetal HR outcomes from a control group (non-SRI treated/non-depressed) with three prenatal exposure groups: fetuses of mothers who were SRI-treated/depressed, SRI-treated/non-depressed, and non-SRI treated/depressed. These groups captured how maternal response to SRI pharmacotherapy, namely whether depressive symptoms persisted or remitted, may differentially influence the fetus. We hypothesized that acute SRI exposure would be associated with reduced fetal HR variability and that SRI-exposed fetuses with concurrent exposure to maternal depressed mood would have the greatest changes compared with outcomes in non-exposed fetuses.

## Materials and Methods

### Study Cohort

The study protocols were approved by the UBC Clinical Research Ethics Board and the BC Women's Hospital Research Review Committee (H05-70629 and H12-00733). During the late second trimester, 188 women with singleton low-risk pregnancies were recruited in two cohorts from the Reproductive Mental Health Clinic at BC Women's Hospital and Health Center, community midwives, or family physicians in metropolitan Vancouver, Canada (from November 2006–January 2010 and March 2013–August 2017). Informed consent was obtained from all participants. Both SRI-treated and non-SRI-treated women were recruited who were experiencing a range of antenatal depressive symptoms, some meeting a diagnostic threshold for a DSM-V mood disorder (65), while others were symptomatic at a subthreshold level or were relatively euthymic. Inclusion criteria for SRI-treated women required the initiation of pharmacotherapy before or during pregnancy for a minimum of 90 days prior to delivery (i.e., entire duration of the third-trimester). Demographic characteristics were collected by clinician interviews and health records chart review. Fetal gestational age was calculated using the first trimester dating scan, as per the Society of Obstetricians and Gynaecologists of Canada Clinical Practice Guidelines (66). Exclusion criteria comprised of maternal psychiatric disorders other than unipolar depression or anxiety, illicit substance use, gestational hypertension or diabetes, placental insufficiency, or any other significant maternal or fetal medical condition. Fetuses born prior to 36-weeks' gestation were excluded.

Of the 188 recruited women, 153 were eligible for inclusion in the present study. Reasons for exclusion were as follows: cancelation for technical reasons (*n* = 12), preterm delivery (*n* = 8), obstetrical complications (*n* = 8), emergent issues during the study protocol necessitating clinical assessment (*n* = 4), voluntary withdrawal (*n* = 2), and development of an exclusion criterion after recruitment (*n* = 1).

Of note, the present study reports on two maternal-fetal cohorts that underwent nearly identical data collection sequences at 36-weeks' gestation, with the exception of maternal blood collection (detailed below) on the first cohort only. These cohorts did not differ in clinical or demographic characteristics. Subsets of data from participants in the present study had been included in two prior reports investigating fetal outcomes in healthy, uncomplicated pregnancies (*n* = 68) (67), and SRI-exposure effects on brain blood flow (*n* = 74) (30). While primary study protocols were similar, the present study investigated acute and chronic effects of SRI exposure in relation to fetal HR variability, maternal pharmacologic data and the potentially confounding effects of depressed mood. These augmented data and outcomes have not been previously reported.

### Maternal Depressed Mood and SRI Antidepressants

Maternal depressive symptoms were assessed with the Hamilton Rating Scale for Depression (HAM-D) (68), a 17-item clinician-rated questionnaire administered by trained research staff, blinded to SRI exposure-status. Mothers were considered to be symptomatically depressed with a total HAM-D score > 8 (69). In this study, SRI antidepressants included any selective serotonin reuptake inhibitor (SSRI) or serotonin-norepinephrine reuptake inhibitor (SNRI).

To detect SRI-related fetal effects and distinguish them from exposure to maternal depressed mood, mothers were then grouped based on SRI treatment and the presence of depressive symptoms at 36-weeks' gestation, yielding four study groups: SRI-Depressed (SRI-treated + HAM-D > 8, i.e., depressive symptoms persisted), SRI-Non-Depressed (SRI-treated + HAM-D ≤ 8, i.e., depressive symptoms remitted), Depressed (non-SRI-treated + HAM-D > 8), and Control (non-SRI-treated + HAM-D ≤ 8). Thus, fetal outcome was assessed as an exposure to one of these groups.

### Study Protocol

**Figure 1:**
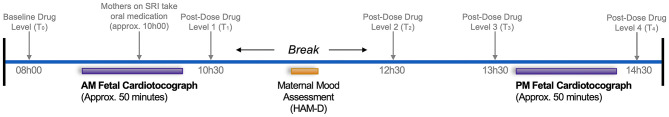
Data collection sequence at 36-weeks' gestation assessing pre- and post-SRI dose effects on the fetus. Text in gray pertains to SRI-treated mothers only. Times are approximate and represent the median, rounded to the nearest 30-min.

Figure 1 outlines the fetal and maternal data collection sequence that occurred at 36-weeks' gestation. On the day of the study, all participants were instructed to eat and drink as per usual prior to arrival. Participants underwent two sequential fetal assessments in a dedicated quiet room at the BC Women's Hospital Center for Prenatal Diagnosis, first in the morning (AM/baseline; ~09h30) and again in the afternoon (PM; ~13h30); methodological details are described below. Mothers were positioned in the left recumbent position to prevent aortocaval compression. Fetal assessments were separated by a 2-h controlled break, involving the administration of the HAM-D and time for participants to mobilize and have lunch (provided).

To investigate chronic and acute SRI effects on the fetus, SRI-treated women were asked to withhold their typical morning oral dose until ~10h00, resulting in the AM/baseline and PM fetal assessments corresponding to pre-dose and post-dose periods, respectively. To characterize concurrent SRI pharmacokinetics across the study protocol, plasma drug concentrations were quantified at baseline (pre-dose) and four time-points post-dose; details on the drug level assay and pharmacologic variables are described below. Timing for each component of this study considered the need for a sufficient antidepressant baseline (pharmacologic trough), half-life, and time-to-peak plasma levels, weighted against length of study in effort to minimize maternal discomfort/inconvenience and potential effects of diurnal variations in the fetal variables obtained.

### Fetal Cardiotocography

Fetal cardiotocography (CTG) was used to investigate patterns of fetal HR and HR variability. Fetal HR was recorded continuously for 50-min using a *Sonicaid Fetal Care* computerized CTG system (*Huntleigh Healthcare Ltd*.; Cardiff, UK; software version 2.2.3.0), a clinical tool widely used for antenatal fetal surveillance (70). Briefly, the software baseline-fits the continuous fetal HR tracing then computes several variables based on its averaging algorithm (71, 72): *basal fetal HR* (i.e., average resting HR, in beats per minute; bpm), number of fetal HR *accelerations* and *decelerations*, as well as three measures of fetal HR variability: *short-term variation* (STV), *high variability* and *low variability*. STV, a measure of micro-fluctuations in fetal HR, was computed as the average epoch-to-epoch variation across the entire HR tracing in pulse intervals (i.e., time between consecutive heart beats, in milliseconds; ms). Whereas high and low variability reflect specific HR patterns that occur during periods of fetal activity and quiescence, respectively. Episodes of high and low variability were computed as the sum of all individual episodes (in minutes) each HR pattern was displayed in the tracing, corrected to 50-min. Additionally, the number of maternally-perceived fetal movements (FMs) during each CTG was recorded using a handheld event marker, which we assessed as an indirect measure of fetal motor activity. Refer to Pardey et al. for further details on reported measures (72).

### Maternal SRI Plasma Levels

Changes in maternal plasma drug concentration between fetal assessments were determined by analyzing blood samples from SRI-treated mothers pre-dose (T_0_, baseline levels; ~08h00) and at four time-points post-dose: T_1_ (~10h30), T_2_ (~12h30), T_3_ (~13h30), and T_4_ (~14h30). Serum was separated by centrifugation at 3,000 × g for 10 min, transferred to polypropylene tubes and stored at −70°C until analysis. High performance liquid chromatography tandem mass spectrometry, performed offsite (CANTEST Ltd.; Burnaby, Canada), was used to determine levels of fluoxetine, norfluoxetine, paroxetine, sertraline, citalopram, escitalopram and venlafaxine. The calibration range was 0.1–100 ng/ml for analytes (except sertraline, where the lower limit of quantification was 0.25 ng/ml). The intra- and inter-assay coefficients of variation and relative errors were <20% for all drugs and metabolites.

Plasma drug concentrations were adjusted for maternal oral dose (ng/ml·mg). To quantify the relative change in maternal SRI level between the pre- and post-dose fetal assessments, the difference in dose-adjusted plasma drug concentration between T_0_ and T_3_ was determined. Plasma concentrations for metabolites were not reported as they reflect parent drugs.

#### SRI Pharmacokinetics and Standardized Dose

Maternal SRI plasma levels were further characterized by performing a non-compartmental pharmacokinetic analysis, yielding estimates of maximum plasma drug concentration (C_max_), time-to-peak (T_peak_) and area under the curve (AUC_last_). Pharmacokinetic variables were calculated using the *PKNCA* R package (73).

Further, we computed a standardized SRI dose variable to investigate whether dose-dependent relationships were present among SRI-related fetal outcomes. As per methods described by Mulder et al. (28), standardized SRI dose was defined according to the World Health Organization Anatomical Therapeutic Chemical-Defined Daily Dose (ATC-DDD) Index (74). DDDs were as follows: 10 mg for escitalopram; 20 mg for citalopram, fluoxetine, and paroxetine; 50 mg for sertraline; 100 mg for venlafaxine; and 300 mg for moclobemide. Mothers prescribed their antidepressant's DDD were set to 1; higher or lower doses were expressed as a multiple of the DDD.

### Statistical Analyses

Statistical analyses were performed using *R Statistical Computing Environment* version 3.6.1 (75); the significance level was set at α = 0.05. Group differences in maternal and fetal characteristics were assessed using one-way analysis of variance (ANOVA) or Kruskal-Wallis rank sum test for continuous normal and ordinal data, respectively; significant between-group effects were further explored using *post hoc* Tukey's HSD or the Dunn test. Chi Square tests were used for group comparisons of categorical variables.

Generalized linear mixed-effects models (GLMMs) were used to investigate group differences in fetal HR and HR variability outcomes across time. GLMMs describe each outcome as a linear combination of fixed and random effects; here, fixed effects were an interaction between one between-factor (*Group*: Control, Depressed, SRI-Depressed, SRI-Non-Depressed) and one within-factor (*Time*: AM/pre-dose, PM/post-dose). Gestational age at the time of assessment and fetal sex were also included as fixed effects terms. Because pre- and post-dose outcomes were not independent, random effects were specified to account for individual differences at baseline (AM/pre-dose; i.e., random intercept for subjects) and the within-subject variability explained by the repeated measures (i.e., random slope for subjects across *Time*) (76). Linear or Poisson (log) link functions were specified according to the underlying distribution. Mixed modeling was conducted using the *lme4* library in R (77) and fit by restricted maximum likelihood. Type III Wald *F*-statistics (or *X*^2^-statistic, if Poisson model) and associated p-values are reported for significant interaction or main effects; effective degrees of freedom were estimated with the Kenward-Roger approximation.

*Post hoc* tests explored significant *Group* × *Time* interactions to detect group differences at AM/pre-dose and PM/post-dose assessments, as well as within-group changes across time. Results are reported as the estimated difference between relevant factor contrasts, along with 95% confidence intervals (CI) and associated *p*-values, adjusted for multiple comparisons with Tukey's method. Further, given the previous reports of sex differences in fetal HR [e.g. (78)], we also investigated whether any significant effect differed between male and female fetuses. Additional *post hoc* GLMMs examined *Group* × *Sex* × *Time* (three-way) interactions, adjusted for gestational age. *Post hoc* testing was performed using the *emmeans* R package (79).

## Results

Of the 153 mother-fetal participants, 148 were included in the study sample: one SRI-treated mother was not compliant with study protocols, one fetus did not meet the Dawes/Redman criteria for normality during CTG sessions (71, 72), one fetus was found to have a cardiac abnormality, one fetus had overall poor data, and one fetus was consistently an outlier in analysis. The final study cohort comprised 57 Control, 42 Depressed, 31 SRI-Depressed, and 18 SRI-Non-Depressed mother-fetus pairs. Maternal and fetal characteristics did not differ between those included in the analysis sample (*n* = 148) compared to those who did not participate/were excluded (*n* = 40) (Supplementary Table 1), other than in characteristics related to exclusion criteria (i.e., preterm delivery).

### Maternal and Fetal Characteristics

Maternal characteristics generally did not differ between groups (Table 1), apart from maternal weight at 36-weeks' gestation, which was higher in both Depressed (*p*_*adj*_ = 0.05) and SRI-Depressed (*p*_*adj*_ = 0.05) women compared to Controls. Maternal depressed mood symptoms differed between groups, with significantly higher HAM-D scores in the Depressed and SRI-Depressed groups compared to women in both the Control and SRI-Non-Depressed groups (all: *p*_*adj*_ < 0.001). Mood symptoms among SRI-Non-Depressed women did not differ from Controls (*p*_*adj*_ = 0.4).

**Table 1:** Maternal characteristics (*n* = 148).

	**Control**	**Depressed**	**SRI-Depressed**	**SRI-Non-Depressed**	**Test statistic**
	**(*n* = 57)**	**(*n* = 42)**	**(*n* = 31)**	**(*n* = 18)**	**(*p*-value)**
Maternal age (years)	32.9 ± 3.5	34.5 ± 4.5	33.9 ± 5.9	35.1 ± 5.1	*F*_(3, 144)_ = 1.6 (0.2)
Maternal weight at 36-weeks' (kg)	75.1 ± 9.7	81.9 ± 16.0	82.5 ± 15.0	79.6 ± 9.8	*F*_(3, 144)_ = 3.2 (0.02)*
Parity	0 (0, 1)	0 (0, 1)	0 (0, 1)	0 (0, 0)	*H*_(3)_ = 1.9 (0.6)
Education (total years)	18.7 ± 3.1	18.0 ± 3.9	17.3 ± 3.6	18.3 ± 3.7	*F*_(3, 144)_ = 1.1 (0.3)
Alcohol during pregnancy (*n* total drinks)^†^	0 (0, 2)	0 (0, 2)	0 (0, 2.5)	1 (0, 4.75)	*H*_(3)_ = 2.6 (0.5)
Smoking during pregnancy (*n* smoker/*n* non-smoker)	0/57	1/41	1/30	1/17	(0.2)
HAM-D at 36-weeks'	4.7 ± 2.3	12.7 ± 4.0	13.4 ± 3.1	6.0 ± 2.1	*F*_(3, 144)_ = 88 (<0.001)***
**SRI antidepressants (** ***n*** **, [dose range])**					
Citalopram (*n* = 14)	—	—	10 [10–60 mg]	4 [10–50 mg]	—
Escitalopram (*n* = 7)	—	—	3 [5–20 mg]	4 [10 mg]	—
Fluoxetine (*n* = 5)	—	—	2 [20–80 mg]	3 [20–60 mg]	—
Paroxetine (*n* = 4)	—	—	3 [20–40 mg]	1 [30 mg]	—
Sertraline (*n* = 6)	—	—	4 [50–200 mg]	2 [75–200 mg]	—
Venlafaxine (*n* = 12)	—	—	9 [75–262.5 mg]	3 [75–150 mg]	—
Moclobemide^‡^ (*n* = 1)	—	—	—	1 [150 mg]	—
Standardized daily SRI dose	—	—	1.5 (1.0, 2.0)	1.0 (1.0, 1.5)	*t*_(47)_ = 0.9 (0.4)
Length of gestational SRI exposure (days)	—	—	264 ± 36	260 ± 48	*t*_(47)_ = 0.31 (0.8)

Continuous variables reported as mean ± SD if normally distributed, or median (first, third quartile) if skewed. Categorical variable reported as total number (n). Test statistics, degrees of freedom, and associated p-values are reported for between-group differences using: one-way ANOVA (F), Kruskal-Wallis test (H), Fisher's Exact test, or two-sample t-test (t), where appropriate. P-value significance levels: *p < 0.05, **p < 0.01, ***p < 0.001.

*SRI, serotonin reuptake inhibitor; HAM-D, total score from Hamilton Rating Scale for Depression; kg, kilograms; mg, milligrams*.

† *Alcohol during pregnancy represents n total standard drinks consumed during the course of pregnancy (study sample range: 0–52 total drinks)*.

‡ *Reversible monoamine oxidase inhibitors included in cohort as “SRI-exposed.”*

SRI-treated women were taking a daily oral dose within the typical therapeutic range and were prescribed their antidepressant for the entire duration of pregnancy, except four mothers with third-trimester exposure only (i.e., *n* = 4 taking SRI for 137 ± 44 days prior to delivery). Neither standardized SRI dose nor length of gestational SRI exposure differed between SRI-Depressed and SRI-Non-Depressed mothers. Included in the SRI-Non-Depressed group was one mother treated with moclobemide, a reversible inhibitor of monoamine oxidase-A, which also acts to increase serotonergic activity by inhibiting 5-HT deamination within neurons and synaptic vesicles (80).

Fetuses were assessed at 35.9 ± 0.81 weeks' gestation; their characteristics are summarized in Table 2. All fetuses included in analysis were delivered at term and were clinically healthy newborns discharged from hospital according to routine schedules. Gestational age at birth was significantly lower for fetuses in the SRI-Depressed group compared to the Control (*p*_*adj*_ < 0.001) and Depressed (*p*_*adj*_ = 0.002) groups. In the newborn period, the SRI-Depressed group also had lower birth weight, length and head circumference compared the Control and Depressed groups; however, these effects all diminished when adjusting for gestational age at birth.

**Table 2:** Fetal characteristics (*n* = 148).

	**Control**	**Depressed**	**SRI-Depressed**	**SRI-Non-Depressed**	**Test statistic**
	**(*n* = 57)**	**(*n* = 42)**	**(*n* = 31)**	**(*n* = 18)**	**(*p*-value)**
Gestational age at fetal study (weeks)	36.0 ± 0.9	35.9 ± 0.8	35.9 ± 0.7	35.9 ± 0.8	*F*_(3, 144)_ = 0.1 (> 0.9)
Gestational age at birth (weeks)	39.9 ± 1.1	39.8 ± 1.3	38.9 ± 1.2	39.6 ± 1.5	*F*_(3, 144)_ = 5.5 (0.001)**
Sex (*n* male/*n* female)	26/31	26/16	14/17	6/12	*χ^2^*_(3)_ = 5.0 (0.2)
Birth weight (g)	3532 ± 408	3588 ± 416	3312 ± 490	3514 ± 431	*F*_(3, 144)_ = 2.7 (0.05)
Length at birth (cm)	52.0 ± 2.1	51.7 ± 2.3	50.3 ± 1.8	51.4 ± 2.7	*F*_(3, 144)_ = 4.2 (0.007)**
Head circumference at birth (cm)	35.2 ± 1.3	35.1 ± 1.4	34.3 ± 1.4	34.9 ± 1.1	*F*_(3, 144)_ = 3.6 (0.02)*
Apgar at 5 min	9 (9, 9)	9 (9, 9)	9 (9, 9)	9 (9, 9)	*H*_(3)_ = 2.2 (0.5)

Continuous variables reported as mean ± SD if normally distributed, or median (first, third quartile) if skewed. Categorical variable reported as total number (n). Test statistics, degrees of freedom, and associated p-values are reported for between-group differences using: one-way ANOVA (F), Kruskal-Wallis test (H), or Chi Square test (χ^2^), where appropriate. P-value significance levels: *p < 0.05, **p < 0.01, ***p < 0.001.

*SRI, (fetal exposure to) serotonin reuptake inhibitor antidepressant; g, grams; cm, centimeters*.

### Maternal SRI Pharmacokinetics

Plasma drug concentrations were quantified for a minimum of three of the five time-points in 24 of the 49 SRI-treated women. One mother's plasma drug concentrations were below lower levels of quantification (<0.1 ng/ml) at T_0_ and T_1_, and marginally above quantification for remaining pose-dose levels; this subject's drug data were excluded, resulting in a maternal SRI plasma level sample of *n* = 23. Aside from having higher weight at 36-weeks' gestation, these mothers were considered representative of the larger SRI-treated study sample as there were no other differences between those with (*n* = 23) and without (*n* = 26) drug level data (Supplementary Table 2). Refer to Supplementary Table 3 for times of maternal blood collection and corresponding plasma drug level (ng/ml) data.

**Figure 2:**
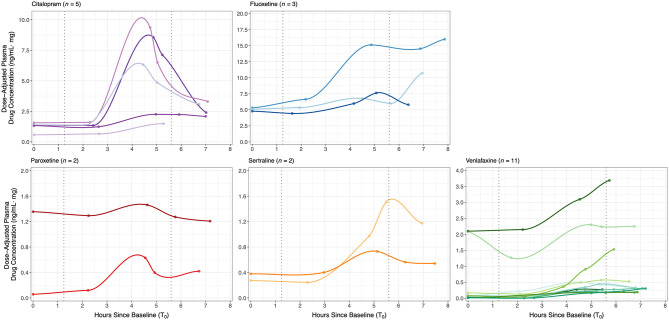
Dose-adjusted plasma drug concentrations (ng/ml·mg) at baseline (T_0_) and four time-points post-dose across the study protocol for 23 SRI-treated mothers, grouped by antidepressant type. Concentration-time curves demonstrate inter-individual variability in SRI pharmacokinetics and maternal drug levels relative to the start of each fetal assessment; curves were fit to each individual's data with local polynomial regression. SRI oral dosing occurred a median of 1.83 h after T_0_. Median blood collection times were: baseline (T_0_) at 08h06, post-dose 1 (T_1_) at 10h21, post-dose 2 (T_2_) at 12h50, post-dose 3 (T_3_) at 13h38, and post-dose 4 (T_4_) at 14h57. Median start times of the baseline/pre-dose and post-dose fetal assessments were at a 1.3 and 5.6 h after T_0_, respectively (dotted vertical lines).

Figure 2 shows the inter-individual variability in concentration-time curves across the study protocol, grouped by antidepressant type. Baseline levels between T_0_ and T_1_ were the lowest plasma concentrations, reflecting a pharmacologic trough at apparent steady-state prior to oral SRI dose, which occurred 1.8 ± 0.3 h after T_0_ and a median of 26 h (interquartile range (IQR): 24–27) since the reported previous dose. Concentration-time curves illustrate an expected increase in plasma drug concertation as part of the absorption phase following oral dose, with individuals on citalopram, paroxetine and sertraline, as well as some individuals on venlafaxine, reaching maximum concentration (C_max_) between 4.5–6 h (T_peak_), followed by the initial elimination phase. Fluoxetine-treated mothers appear to still be in the absorption phase when final drug levels were collected (T_4_), consistent with a T_peak_ of 6–8 h. AUC_last_, representing total observed maternal drug exposure during our study protocol, was highest for fluoxetine and lowest for paroxetine. Maternal pharmacokinetic responses are summarized in Table 3.

**Table 3:** Maternal pharmacokinetic variables (mean ± SE) for each antidepressant type on a subset of SRI-treated women (*n* = 23).

**Antidepressant**	***N***	**AUC_**last**_**	**C_**max**_**	**T_**peak**_**	**Δ T3-T0**
			**(ng/ml)**	**(h)**	**(ng/ml·mg)**
Citalopram	5	906 ± 271	274 ± 92	4.9 ± 0.14	3.2 ± 1.0
Fluoxetine^†^	3	3221 ± 1919	613 ± 342	5.8 ± 1.0	4.3 ± 2.5
Paroxetine	2	125 ± 66	24 ± 5.2	4.6 ± 0.04	0.13 ± 0.21
Sertraline	2	728 ± 256	197 ± 105	5.3 ± 0.18	0.71 ± 0.53
Venlafaxine	11	621 ± 314	163 ± 74	5.7 ± 0.30	0.43 ± 0.13

*AUC_last_, area under the curve from T_0_ (baseline) to the last measured plasma drug concentration (T_4_); C_max_, maximum (peak) plasma drug concentration (ng/ml); T_peak_, time (hours) from baseline to reach C_max_; Δ T3-T0, change in dose-adjusted plasma concentration (ng/ml·mg) from baseline (T_0_) to post-dose 3 (T_3_), representing the change in maternal drug concentration between baseline/pre-dose and post-dose fetal assessments*.

† *Fluoxetine not yet reached maximum plasma concentration at time of last blood collection (i.e., T_4_ estimated as T_peak_). AUC_last_ and C_max_ for fluoxetine may be underestimations*.

#### Validation of Study Design: Fetal Assessments at Pharmacologic Trough and Peak

Figure 2 also illustrates the start times of each fetal assessment relative maternal plasma SRI levels. For the antidepressants studied, the baseline/pre-dose fetal assessment started a median 1.3 h (IQR: 0.99−1.5) after T_0_ and 0.92 h (IQR: 0.87−1.00) before T_1_, which therefore occurred during the period of steady-state pharmacologic trough (T_0_-T_1_). At a median of 5.6 h (IQR: 5.2−6.0) after T_0_, the start of the post-dose fetal assessment corresponded to the late absorption phase or early elimination phase, depending on SRI type. Dose-adjusted plasma drug concentration significantly increased between baseline/pre-dose and post-dose fetal assessments [*n* = 23; paired *t*-test: *t*_(21)_=3.13, *p* = 0.005], with a mean (± SE) change from T_0_-to-T_3_ of 1.54 ± 0.48 ng/ml·mg.

### Fetal HR and HR Variability

Fetal CTG measures (*n* = 148) are presented in Table 4, and were within clinically normative ranges for gestational age (56, 77). AM/pre-dose and PM/post-dose fetal CTG sessions were 50.1 ± 3.0 min with minimal HR tracing signal loss (3.5 ± 5.4 %); neither the duration nor amount of signal loss differed between groups at either fetal assessment.

**Table 4:** Fetal HR, HR variability and movement (*n* = 148).

	**Control** ** (** ***n*** **=** **57)**		**Depressed** ** (** ***n*** **=** **42)**		**SRI-Depressed** ** (** ***n*** **=** **31)**		**SRI-Non-Depressed** ** (** ***n*** **=** **18)**	
**Fetal variables**	**AM**	**PM**	**AM**	**PM**	**AM/pre-dose**	**PM/post-dose**	**AM/pre-dose**	**PM/post-dose**
Basal HR (bpm)^a^	135 ± 7	136 ± 8	134 ± 7	137 ± 8	134 ± 9	138 ± 8	132 ± 9	139 ± 10
HR accelerations (*n*)^b^	14 (11, 18)	16 (13, 20)	16 (11, 20)	15.5 (12, 19)	12 (9, 15)	14 (12, 17)	14 (10, 18)	13.5 (10, 15)
HR decelerations (*n*)	1 (0, 1)	1 (0, 2)	0 (0, 2)	1 (0, 2)	1 (0, 2)	1 (0, 2)	1 (0, 2)	2 (0.25, 3)
STV (ms)^a^	10.4 ± 3.1	10.9 ± 2.9	10.9 ± 3.1	10.7 ± 3.0	10.6 ± 3.4	9.6 ± 2.4	11.5 ± 2.7	9.5 ± 2.1
High HR variability (min)^a^	30.0 ± 13.0	32.5 ± 11.9	31.2 ± 12.1	33.1 ± 11.2	30.3 ± 14.3	31.4 ± 11.0	34.9 ± 9.3	25.7 ± 12.4
Low HR variability (min)	0.97 ± 2.5	1.8 ± 4.1	1.7 ± 4.2	1.4 ± 4.0	1.5 ± 2.9	0.70 ± 2.2	0.50 ± 1.9	1.8 ± 4.4
Fetal movements/hour (*n*)^†^	50 (31, 66)	53 (35, 82)	53.5 (37, 77)	57 (38, 86)	46 (28, 62)	47 (30, 69)	43.5 (30, 83)	60 (32, 83)

*Fetal variables for each group are summarized as mean ± SD if continuous and normally-distributed, or median (first, third quartile) if skewed or count data*.

*GLMM Statistics: significant fixed effects are identified as: ^a^significant Group × Time interaction; ^b^significant effect of Time; and ^c^significant effect of Group. Refer to text for model statistics and estimated marginal means between relevant factor contrasts*.

*AM, morning/baseline fetal assessment; PM, afternoon fetal assessment; HR, heart rate; STV, short-term variation; bpm, beats per minute; n, total number; ms, milliseconds; min, minutes*.

† *Maternally-perceived fetal movements per hour (adjusted from ~50 min)*.

#### Basal Fetal HR

**Figure 3:**
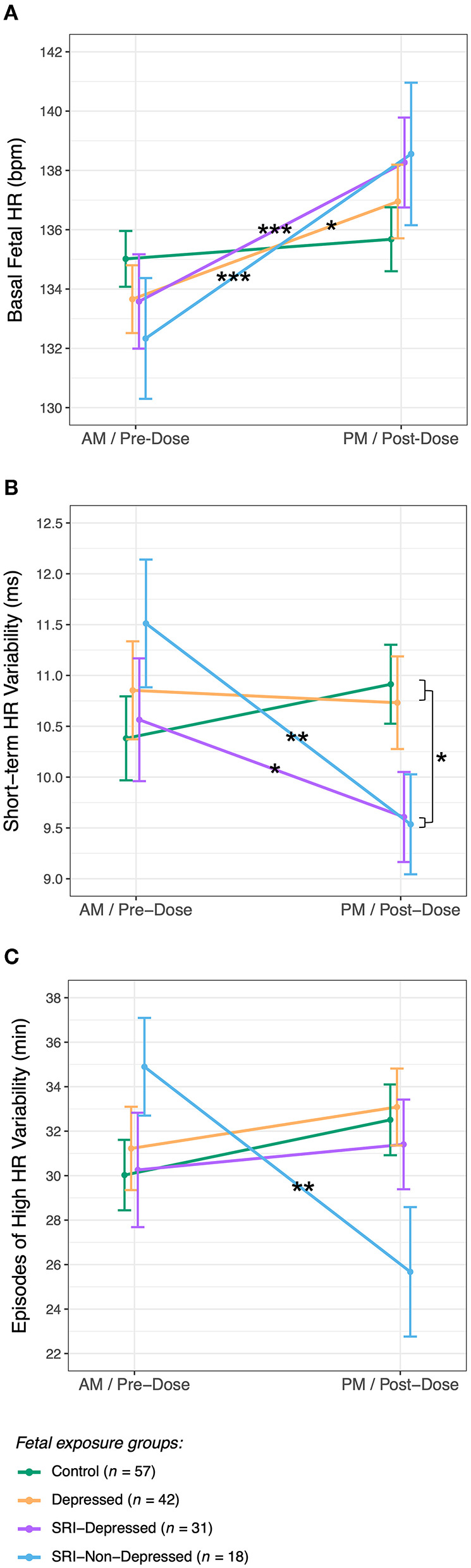
Fetal HR and HR variability (mean ± SE) for each exposure group across AM/pre-dose and PM/post-dose fetal assessments for **(A)** basal fetal HR, **(B)** short-term variability, and **(C)** episodes of high fetal HR variability (*post hoc* test significance levels: *p < 0.05, **p < 0.01, ***p < 0.001).

There were no group differences in basal fetal HR at either fetal assessment. However from AM/pre-dose, fetal HR increased to be significantly higher at the PM/post-dose assessment in all fetal groups, except the Controls (*Group* × *Time* interaction: *F*_(3, 142.0)_ = 3.4, *p* = 0.02) (Figure 3A). Between assessments, basal fetal HR increased by 5 bpm (95% CI: 2.3, 7.7; *p*_*adj*_ < 0.001) in the SRI-Depressed group, by 6 bpm (95% CI: 2.7, 9.8; *p*_*adj*_ < 0.001) in the SRI-Non-Depressed group, and by 3 bpm (95% CI: 0.8, 5.4; *p*_*adj*_ = 0.01) in the Depressed group. In contrast, fetal HR in the Control group did not change between assessments (*p*_*adj*_ = 0.4). There were no covariate effects on basal fetal HR.

#### Fetal HR Accelerations and Decelerations

Fetuses had a median of 14 HR accelerations (IQR: 11–18) and 1 HR deceleration (IQR: 0–2) during each 50-min CTG session (Table 4). Fetal HR accelerations did not differ between groups at either assessment; however, averaged across groups, the number of HR accelerations significantly increased relative to AM/pre-dose assessment (main effect of *Time*: *X*${}_{1}^{2}$ = 8.9, *p* = 0.003). Whereas, fetal HR decelerations did not significantly differ between groups nor across time, but were found to be positively associated with gestational age (*X*${}_{\left(1\right)}^{2}$ = 4.2, *p* = 0.04).

#### Short-Term HR Variability

STV, reflecting the average HR variation across each CTG tracing, in pulse intervals (ms) (72), did not differ between groups at the AM/pre-dose fetal assessment. Following SRI dose, a significant decrease in STV was observed relative to baseline among fetuses in both SRI-exposed groups (*Group* × *Time* interaction: *F*_(3, 142.6)_ = 5.1, *p* = 0.002) (Figure 3B): STV decreased by 1.0 ms (95% CI: 0.05, 1.9; *p*_*adj*_ = 0.04) in SRI-Depressed group fetuses and by 2.0 ms (95% CI: 0.80, 3.2; *p*_*adj*_ = 0.001) in SRI-Non-Depressed group fetuses. These post-dose decreases resulted in SRI-exposed fetuses to have 1.1 ms (95% CI: 0.80, 3.2; *p*_*adj*_ = 0.04) lower STV compared to non-exposed fetuses at the PM/post-dose assessment, controlling for covariates.

#### High and Low Fetal HR Variability

Episodes of high fetal HR variability did not differ between groups at the AM/pre-dose fetal assessment; however post-SRI dose, a *Group* × *Time* interaction was identified (*F*_(3, 142.9)_ = 3.7, *p* = 0.01), whereby the time fetuses in the SRI-Non-Depressed group spent displaying high HR variability decreased by 9.2 min (95% CI: 3.0, 15.4; *p*_*adj*_ = 0.004), controlling for covariates (Figure 3C). No between-group differences were found for episodes of low HR variability.

#### Fetal Motor Activity

Fetal movements (FMs), which did not differ between groups, occurred at a median frequency of 48 (IQR: 32–70) and 53 (IQR: 32–83) movements/hour during the AM/pre-dose and PM/post-dose assessments, respectively (Table 4). As expected, the number of FMs per minute during episodes of high HR variability was significantly higher than during low HR variability (*t* = 6.8, *p* < 0.001); this also did not differ between groups, nor across assessments. There were no covariate effects on FMs.

### Sex-Specific Fetal SRI Effects

**Figure 4:**
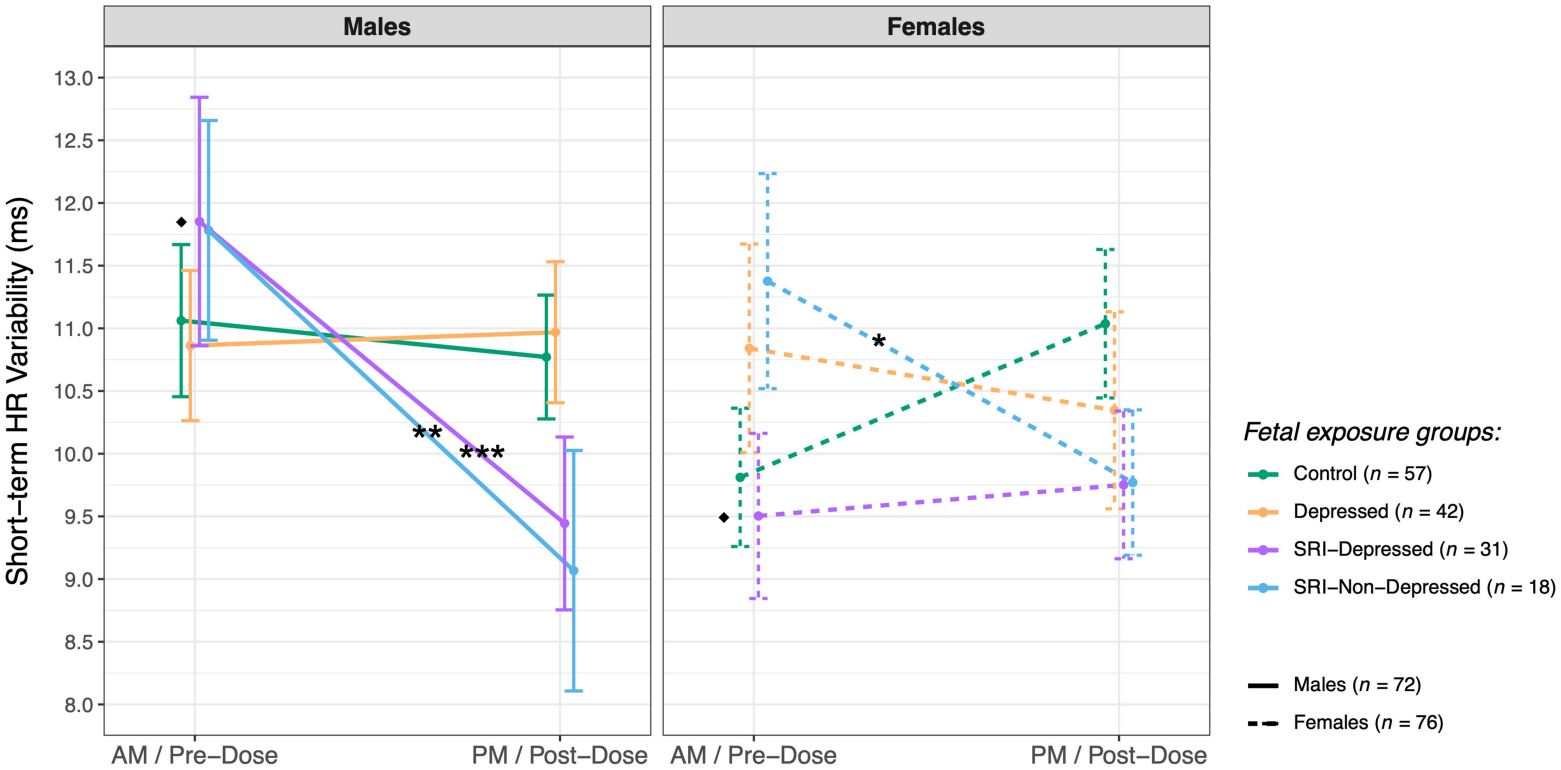
Sex differences in fetal short-term HR variability (mean ± SE) between exposure groups (*post hoc* test significance levels: *p < 0.05, **p < 0.01, ***p < 0.001; *indicates within-sex group difference, ^♦^ indicates within-group sex difference).

*Post hoc* analysis revealed sex-specific effects on group differences in fetal STV (three-way interaction: *F*_(3, 138.4)_ = 2.7, *p* = 0.04) (Figure 4). In the SRI-Depressed group, only male fetuses underwent a significant post-dose decrease in STV: from pre- to post-dose assessments, STV decreased in SRI-Depressed group males by 2.4 ms (95% CI: 1.1, 3.7; *p*_*adj*_ < 0.001), in SRI-Non-Depressed group males by 2.7 ms (95% CI: 0.75, 4.7; *p*_*adj*_ = 0.008), and in SRI-Non-Depressed group females by 1.6 ms (95% CI: 0.22, 3.0; *p*_*adj*_ = 0.02). Conversely, female fetuses in the SRI-Depressed group did not undergo this post-dose decrease in STV, but instead, were found to have 2.2 ms (95% CI: 0.13, 4.4; *p*_*adj*_ = 0.04) lower STV than SRI-Depressed males at the baseline/pre-dose assessment and remained unchanged post-dose. There were no other significant effects of fetal sex on group differences reported.

### SRI Dose-Dependent Fetal Effects

**Figure 5:**
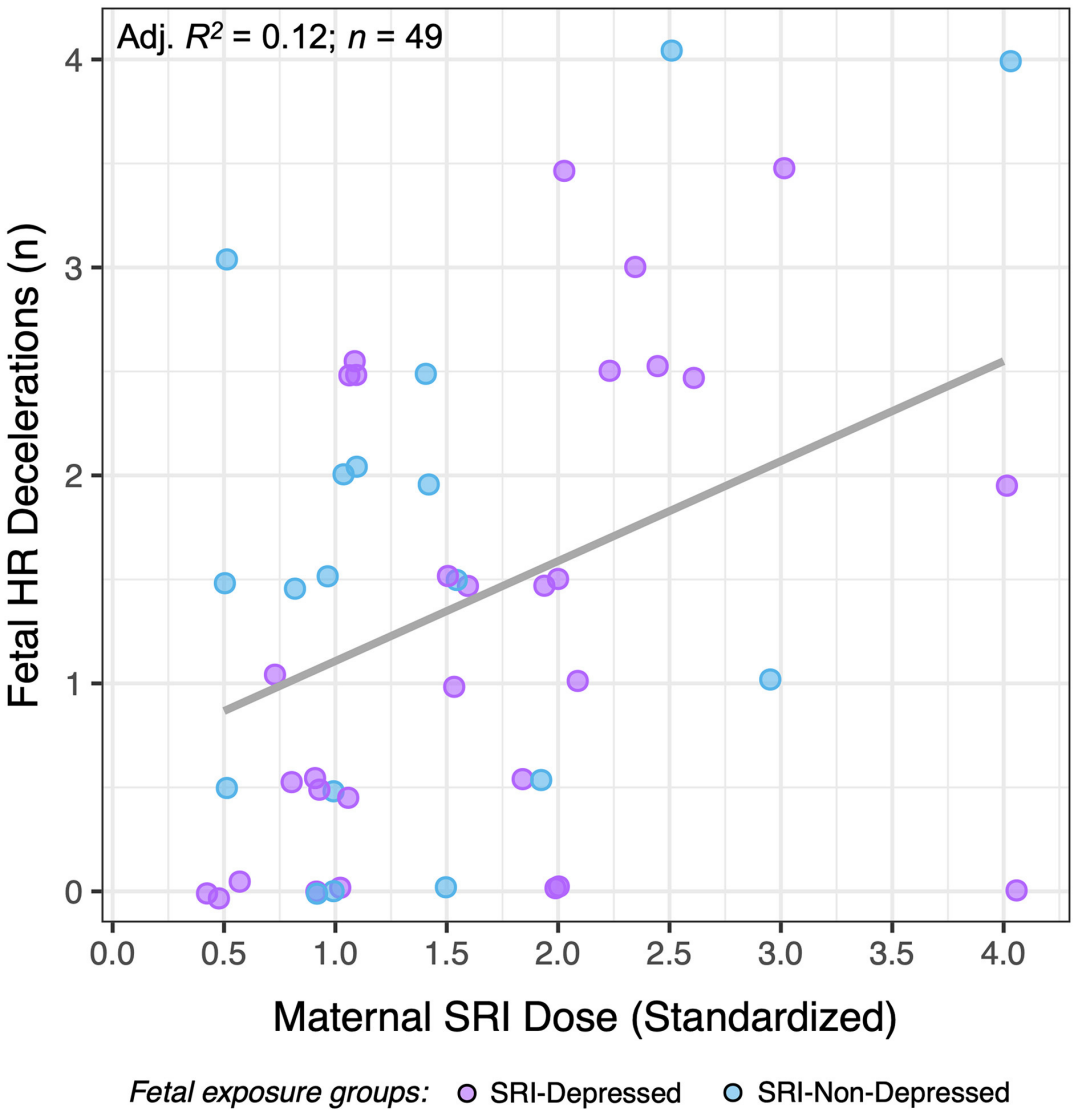
The relationship between standardized maternal SRI oral dose and the number of fetal HR decelerations for SRI-exposed fetuses (SRI-Depressed and SRI-Non-Depressed groups). Data presented represent average HR decelerations across 50-min CTG sessions, which did not differ across time.

Maternal SRI oral dose (standardized) was found to be significantly associated with the number of fetal HR decelerations during 50-min CTG sessions (Figure 5). Higher SRI doses were associated with a greater number of fetal HR decelerations (*n* = 49; *F*_(1, 47)_ = 7.6, *p* = 0.008). This did not differ between SRI-exposure groups, and effects were evident at both pre- and post-dose assessments. No other dose-dependent effects were observed in SRI-related fetal HR outcomes we report, nor were there differences related to antidepressant class (i.e., SSRIs vs. SNRIs).

## Discussion

This study reports three key findings. In late gestation, fetal SRI exposure was associated with: (1) post-dose decreases in fetal HR variability, (2) sex-specific fetal HR outcomes, and (3) concurrent changes in maternal drug levels that reflected pharmacologic trough/peak (acute) periods. Fetal HR increased while fetal HR variability decreased in SRI-exposed fetuses relative to the AM/pre-dose assessment, reflecting an acute effect of SRI exposure. Importantly, fetal outcomes varied depending on maternal response to SRI pharmacotherapy; namely whether the mothers' depressed mood remitted or remained symptomatic (i.e., SRI-Non-Depressed, SRI-Depressed). In particular, STV acutely decreased among fetuses in both SRI-Depressed and SRI-Non-Depressed groups, thus occurring independent of concurrent maternal mood; whereas, high HR variability was found to acutely decrease only among fetuses in the SRI-Non-Depressed group. SRI-related sex differences in fetal HR variability also varied with maternal mood context, with differences between male and female fetuses observed only in the SRI-Depressed group. Further, higher maternal SRI doses were associated with a greater number of fetal HR decelerations across both study periods. Since neither standardized dose nor length of gestational SRI exposure differed between SRI-Depressed and SRI-Non-Depressed women, fetal outcomes in these groups may be acute drug exposure-related effects. In particular, we did not observe group differences pre-dose during a period of pharmacologic trough, which would have reflected a chronic/sustained effect of SRI exposure.

Importantly, changes in fetal HR variability we report were within normative ranges for healthy typically developing fetuses at 36-weeks' gestation (56, 78), and thus, are likely not clinically significant. However, even within a normative range of fetal physiology, we observe group differences that may reflect adverse developmental effects of SRI exposure before birth that vary with respect to the timing of maternal oral dose.

### Fetal SRI Exposure at Pharmacologic Trough and Peak

Maternal SRI plasma concentrations increased following a typical daily oral SRI dose in the third-trimester, demonstrating an expected concentration-time relationship. Women in this study were on long-term SRI pharmacotherapy (most prior to conception) and appear to have trough plasma SRI levels consistent with a pharmacologic steady-state, which were similar to third-trimester maternal dose-adjusted plasma trough levels previously reported (81). Although sample size was limited, women taking citalopram, sertraline, and paroxetine reached C_max_ and were in the early elimination phase at the post-dose fetal assessment. In contrast, women taking venlafaxine and fluoxetine were still in the absorption phase by the last blood collection. Trough and peak levels are an accepted phenomenon for multiple oral dosing regimens, but the critical finding from this study was that the extremes of trough/peak observed translated to a variable fetal response.

Our findings demonstrate that the fetus may experience a chronic exposure to steady-state plasma SRI levels, but subject to continual fluctuations in such exposure with respect to maternal oral dose across a typical day in late gestation. Although this does not directly indicate that equivalent drug changes in fetal circulation occur, changes in maternal SRI plasma levels would have implications toward factors that may impact the extent of fetal SRI exposure. SRIs have high placental permeability (46, 82), and in rodents, fetal citalopram exposure was found to exceed that of the mother 2-h after maternal drug administration (83). Beyond transplacental drug transfer, several other factors could also influence SRI pharmacology in this setting, such as genetic variations in maternal metabolic enzymes (further discussed below), fetoplacental metabolism and clearance, or exposure to other pharmacologic agents (41, 42, 84, 85). Hence, it is almost certain that fetal SRI exposure is not consistent and the distinct acute SRI-related outcomes we report suggest a differential fetal sensitivity may exist to varying maternal SRI plasma levels and/or acute physiologic changes secondary to SRI exposure.

### Fetal HR Variability Decreases Following Acute SRI Exposure

Transient reductions in fetal HR variability with acute SRI exposure may indicate impairments in autonomic functioning. The Dawes-Redman parameter of STV, a standardized clinical marker of perinatal compromise (56, 72, 86), not only summarizes overall HR variation, but may also be a surrogate for fetal sympathovagal regulation. In a comparative study by Seliger et al., CTG-derived STV was highly correlated with the standard deviation of normal-to-normal beat intervals, as well as HR in the low frequency power spectra (87). These indices are commonly obtained from fetal electrocardiography, which has higher temporal resolution than ultrasound-based CTG (i.e., ability to detect QRS complexes in continuous cardiac signal) and are among parameters widely used to examine sympathetic and integrative sympathovagal-mediated HR fluctuations (88). Thus, acute decreases in STV, accompanied by acute increases in basal fetal HR we report in SRI-exposed fetuses, may be consistent with sympathetic activation and/or autonomic withdrawal leading to diminished HR variability.

In adults, reduced HR variability is associated with major depressive disorder (89, 90); however, these effects appear to be strongly mediated by antidepressants (91, 92). Additionally, higher SRI doses may have cardiac side effects in adults, such as QT interval prolongation (93). However, to our knowledge, only two other groups have assessed fetal HR variability in relation to prenatal SRI exposure (28, 31), who each described fetal cardiac patterning using differing methodology, consequently limiting direct comparison with our findings. Critically, neither study reported fetal outcome with respect to the timing of maternal SRI oral dose, so it is unknown whether previous findings reflect fetal outcomes of chronic or acute SRI exposure and may be why no SRI effect on fetal HR/variability was observed in Gustafsson et al. (31). Despite these methodological differences, our findings may have consistencies with disrupted neurobehavioral state previously reported in the near-term fetus by Mulder et al. (28). These effects may reflect altered fetal autonomic functioning, particularly given the roles of serotonin as a neuromodulator of autonomic pathways (94, 95). In the postnatal period, altered cardiac autonomic function following an acute noxious event (phenylketonuria heel lance) was observed in both 2–3 day-old neonates (7) and infants at 2-months of age (8) with prenatal SRI exposure. Additional studies are needed to further characterize acute SRI-related changes in fetal HR variability and determine to what extent such changes exert a fetal programming effect on long-term neurodevelopmental outcome in stress-reactivity, emotion/affective processes, and self-regulation.

Importantly, our findings suggests that maternal mood response to SRI pharmacotherapy may be a key modifier of fetal outcome. However, it remains unknown as to why fetuses of SRI-treated mothers whose depressive symptoms remitted would uniquely display acute reductions in high HR variability: a cardiac pattern that, when coupled with HR accelerations and movement, occurs during periods of active neurobehavioral states (72). Although our study did not assess patterns of fetal motor activity, previous studies have identified fetal state based on HR variability alone [e.g. (96, 97)]. Given the high incidence of concordance between fetal HR and motor activity by 32-weeks' gestation (52), it is conceivable that reduced episodes of high HR variability may indicate fewer and/or shorter periods of active states among fetuses in the SRI-Non-Depressed group, possibly reflecting acute impairments or delayed development. Our findings highlight the need for future studies focused on how SRIs interact with maternal mood to influence fetal autonomic functioning and neurobehavior.

### Acute SRI Effects on Fetal HR Variability Are Sex-Specific

Acute SRI-related outcomes in fetal HR variability were found to be moderated by fetal sex. Specifically, the post-dose decrease in STV was observed among SRI-Depressed group male fetuses, compared with the relative stability in STV between assessments in SRI-Depressed females. Indeed, sex difference in fetal HR variability have been reported in low-risk singleton pregnancies (98, 99), for example in a large CTG study, males had lower baseline HR but higher STV than females throughout gestation (78). Although basal HR and STV in males and females in the Control group did not differ significantly, SRI-Depressed males did have higher STV than SRI-Depressed females at the baseline/pre-dose assessment, pointing to a chronic/sustained SRI-related sex difference that is evident when maternal depressive symptoms persist. Sex differences were not observed in the SRI-Non-Depressed group, further suggesting that sex-specific SRI effects vary with maternal mood. Several rodent studies report sex-specific neurodevelopmental outcomes following perinatal SRI exposure, with outcomes that vary with maternal stress/psychiatric context (100). For example, hippocampal neurogenesis and plasticity appear to have a particular sex-specific sensitivity to SRIs and maternal stress (101); interestingly, hippocampal-brainstem connectivity has critical integrative roles in vagal modulation of cardiovascular function (102). In humans, studies reporting sex-specific infant or child outcomes following prenatal SRI exposure are extremely scarce; however, Erickson et al. report that male and female infant temperament trajectories from 3–10 months are differentially associated with prenatal SRI exposure and maternal internalizing symptoms (103), and recently, we identified sex-specific alterations in brain microstructure in neonates with prenatal SRI exposure (104). Moreover, sex differences may influence pharmacologic factors contributing to the extent and effect of SRI exposure on the fetus, such placental functioning (23), metabolic enzyme activity and synaptic transmission (105). While our findings provide the first preliminary evidence that sex-specific SRI effects may emerge in the fetal period with outcomes varying with maternal mood, this topic warrants further investigation in a larger sample.

### Maternal SRI Pharmacology

High inter-individual variability in maternal SRI plasma concentrations was observed in this study, particularly in the concentration-time curves. These differences are indicative of the known population-level heterogeneity in pharmacokinetic factors, likely compounded by pregnancy-induced physiologic changes that influence drug disposition, such as increased gastrointestinal motility, plasma volume, cardiac output and renal function (106). In particular, hepatic cytochrome P450 (CYP) enzymes, which metabolize SRIs, have altered expression and activity across gestation (107, 108). CYP450s are highly polymorphic with high-to-low activity allelic variants (109) and have been associated with individual differences in drug disposition and treatment outcome (110); thus without genetic screening, antidepressant levels will vary widely and unpredictably (111). Indeed, variations in *CYP2D6* genotype are reported to have divergent effects on maternal plasma levels and SRI efficacy during pregnancy (112), which may partially explain why over 60% of our SRI-treated sample remained symptomatic.

Additional factors may also contribute to variable antidepressant efficacy, such as history and initial severity of mental illness, treatment compliance, and other neurobiological factors associated with the pathophysiology of depression and/or antidepressant mechanisms, such as individual differences in synaptic transmission in multiple brain regions (105), genetic expression and endogenous signaling molecules [e.g., reviewed in (113)]. For example, polymorphisms in the serotonin transporter gene promoter (5-HTTLPR) are associated with antidepressant efficacy (114). Emerging evidence also suggests that microRNAs may have regulatory roles in psychological stress pathways, with potential to serve as biomarkers for monitoring antidepressant treatment response (115, 116). In this study, the extent to which each SRI-treated woman experienced symptom remission—or relapse, possibly due to increased maintenance dose requirements with advancing gestation (81)—remains unknown. Future studies combining extended mental health histories with genetic screening, use of novel biomarkers, etc. are needed to elucidate why some women and not others benefit from prenatal SRI treatment, and by extension, how this impacts fetal development.

### Limitations

We note several key limitations pertaining to sample size, study design and methodology in this study. First, sample sizes of fetal exposure groups were relatively small, especially when assessing sex differences. Thus, our findings should be replicated to determine their generalizability. We were also unable to determine whether acute fetal SRI effects were related to specific antidepressants, although we found that fetal outcomes did not differ between antidepressant classes (i.e., SSRIs vs. SNRIs). Further, maternal blood was collected on a subsample of participating women, resulting in particularly small numbers for the SRI pharmacokinetic analysis. Inherent differences in bioavailability and half-life between formulations, along with other factors influencing SRI pharmacokinetics (as discussed above), limited our ability to pool dose-adjusted concentrations for analysis. Between a lack of pooling, small sample size and limited time-frame for sampling (7–8 h), relationships between maternal pharmacokinetic variables (i.e., AUC_last_, C_max_, T_peak_) and SRI-related fetal outcomes were not identified. Future work should investigate whether pharmacokinetic variables, or other biomarkers, may be predictive of acute or chronic fetal outcome as routine blood sampling is rapid and economical.

Our use of four prenatal exposure groups allowed for the distinction between SRI-related fetal HR outcomes from those related to maternal depressed mood, thereby addressing the key methodological constraint of “confounding by indication”. While this approach identified appropriate exposure groups, the impact of maternal depressive illness severity, or variations in symptoms across pregnancy, could not be addressed. Moreover, women scoring close to the depressed/non-depressed cut-off may not differ in a clinically meaningful manner, even though a HAM-D score > 8 (as used here) has been clinically validated as a cut-off between symptomatic and asymptomatic depression (69). However, our findings suggest a differential fetal sensitivity may exist in the context of maternal response to SRI treatment, highlighting the importance of making such distinctions in future studies.

Regarding study design limitations, it is possible diurnal rhythms in fetal cardiovascular variables [e.g., (117, 118)] were an unmeasured source of variability. Even with effort to minimize diurnal effects with the careful consideration of timing for each component of this study, such influences may be driving the increase in basal fetal HR and HR accelerations observed between assessments. It is also possible maternal mood and/or antidepressant treatment may impact maternal circadian cycles, to which the developing fetus may be sensitive (119, 120). Further, with a cross-sectional approach at 36-weeks' gestation, these findings are only relevant to the late gestation fetus and may not reflect changes across earlier periods of prenatal development. Future studies should determine if other aspects of fetal physiology or neurodevelopment demonstrate varying chronic/acute outcomes with respect to maternal SRI dosing.

Lastly, key methodological limitations should also be considered. Doppler-based detection of continuous fetal HR with CTG suffers from low temporal resolution compared to more sophisticated tools, such as fetal electrocardiography or magnetocardiography that can be used for complex HR variability analyses and resolving fast vagal activity (121). However, fetal CTG is widely accessible, cost-effective, and does not require a specialist to administer, thereby aiding in reproducibly. Our findings may also have clinical implications, as fetal CTG measures are implemented in national guidelines for antenatal fetal monitoring (70). Another methodological limitation was the measure of fetal motor activity by maternal perception. Although this provides a crude index of relative fetal activity during the assessment period, many factors can influence maternal perception of her fetus, such as BMI, levels of maternal activity, and the size/growth rate of the fetus (50, 122). As such, the lack of independently recorded fetal movement data limited our ability to separately assess fetal neurobehavioral state from HR tracings. Although it is possible acute decreases in HR variability may reflect variations in fetal state, future studies are needed to investigate fetal HR-movement coupling in this context.

## Conclusions

Prenatal SRI antidepressant exposure had acute, but not chronic, effects on fetal HR and HR variability in late gestation, which differed depending on maternal mood response to SRI pharmacotherapy. Maternal SRI pharmacokinetics had high inter-individual variability, but suggested that fetal SRI exposure is inconsistent and may be sensitive to periods of chronic (trough) and acute (peak) maternal SRI levels. This study also identified sex-specific fetal SRI effects, as SRI-exposed male fetuses displayed post-dose decreases in fetal HR variability, whereas outcomes in SRI-exposed females varied with maternal depressed mood. It remains to be determined whether acute SRI-related decreases in fetal HR variability reflect a transient impairment in fetal autonomic functioning, a pharmacologic mechanism on fetal cardiac patterning, or an *in utero* adaption to long-term altered serotonergic signaling. While replication is needed, these findings may have potential clinical implications for antenatal fetal monitoring and may ultimately improve understanding of developmental risk associated with maternal psychotropic medication use during pregnancy. Future work will investigate longitudinal relationships with postnatal outcomes in infant temperament, stress-regulation and broader neurobehavior, and the manner in which maternal SRI pharmacology, psychiatric distress, and other factors, such as sex, interact to exert a fetal programming effect.

## Data Availability Statement

The raw data supporting the conclusions of this article will be made available by the authors, without undue reservation.

## Ethics Statement

The studies involving human participants were reviewed and approved by UBC Clinical Research Ethics Board. The patients/participants provided their written informed consent to participate in this study.

## Author Contributions

KC aided in data acquisition, performed all data processing and statistical analyses, interpreted the findings, and prepared the manuscript. AC interpreted pharmacologic findings and contributed to manuscript writing. MI contributed to statistical analyses. UB facilitated participant recruitment/retention and aided in data acquisition. DR, TO, and KL conceptualized the study design, obtained funding, interpreted findings, and contributed to manuscript writing. All authors read, critically revised and approved the submitted manuscript.

## Conflict of Interest

The authors declare that the research was conducted in the absence of any commercial or financial relationships that could be construed as a potential conflict of interest.

## Publisher's Note

All claims expressed in this article are solely those of the authors and do not necessarily represent those of their affiliated organizations, or those of the publisher, the editors and the reviewers. Any product that may be evaluated in this article, or claim that may be made by its manufacturer, is not guaranteed or endorsed by the publisher.
