# Supplementary material for: Maternal Serotonin Reuptake Inhibitor Antidepressants Have Acute Effects on Fetal Heart Rate Variability in Late Gestation
Source: Front Psychiatry. 2021 Aug 16;12:680177. doi: 10.3389/fpsyt.2021.680177 (PMC8415315; doi:10.3389/fpsyt.2021.680177)
Supplement: Supplementary file 1 [file Table_1.docx]

Supplementary Material

**Supplementary Table 1:** Comparison of maternal and fetal characteristics between those included in the study sample (*n* = 148) and those who did not participate/were excluded (*n* = 40).

|  | **Study Sample**  **(*n* = 148)** | **Not Studied / Excluded (*n* = 40)** | **Test Statistic**  **(p-value)** |
| --- | --- | --- | --- |
| ***Maternal Characteristics*** |  |  |  |
| Group (Ctl / D / S-D / S-ND) | 57 / 42 / 31 / 18 | 13 / 12 / 10 / 5 | *χ^2^*_(3)_ = 0.58 (<0.9) |
| Maternal Age (years) | 33.9 ± 4.6 | 35.3 ± 4.8 | *t*_(182)_ = 1.6 (0.1) |
| Maternal Weight at 36 weeks (kg) | 79.1 ± 13.2 | 84.5 ± 15.2 | *t*_(175)_ = 1.9 (0.05) |
| Parity (*n*) | 0 (0, 1) | 0 (0, 1) | *W* = 2429 (0.4) |
| Education (years) | 18.2 ± 3.5 | 17.8 ± 3.1 | *t*_(184)_ = 0.53 (0.6) |
| Alcohol During Pregnancy  (*n* total drinks) | 0 (0, 2.25) | 0 (0, 1) | *W* = 2570 (0.3) |
| HAM-D score (at 36-weeks’) | 9.0 ± 5.0 | 10.4 ± 5.3 | *t*_(171)_ = 1.3 (0.2) |
| ***Fetal Characteristics*** |  |  |  |
| Gestational Age at Fetal Study (weeks) | 35.9 ± 0.8 | 35.8 ± 1.3 | *t*_(179)_ = 0.81 (0.4) |
| Gestational Age at Birth (weeks) | 39.7 ± 1.3 | 38.2 ± 2.4 | *t*_(182)_ = 5.2 (<0.001) |
| Sex (Male / Female) | 72 / 76 | 21 / 15 (4 NA) | *χ^2^*_(1)_ = 0.73 (0.4) |
| Birth Weight (g) | 3499 ± 438 | 3170 ± 681 | *t*_(182)_ = 3.6 (<0.001) |
| Length at Birth (cm) | 51.5 ± 2.2 | 50.3 ± 2.9 | *t*_(181)_ = 2.5 (0.01) |
| Head Circumference at Birth (cm) | 34.9 ± 1.4 | 34.4 ± 1.8 | *t*_(181)_ = 2.1 (0.04) |
| Apgar at 5 min | 9 (9, 9) | 9 (8, 9) | *W* = 2240 (0.03) |

*Continuous variables reported as mean ± SD if normally distributed, or median (first, third quartile) if skewed. Categorical variables reported as total number (n). Test statistics, degrees of freedom, and associated p-values are reported for between-group differences using the two sample t-test (t), Chi Square test (χ^2^), or Wilcoxon rank sum test (W), where appropriate.*

*Ctl, Control group; D, Depressed group; S-D, SRI-Depressed group; S-ND, SRI-Non-Depressed group.*

*SRI, serotonin reuptake inhibitor; HAM-D, total score from Hamilton Rating Scale for Depression; kg, kilograms; g, grams; cm, centimeters; NA, Not Available.*

**Supplementary Table 2:** Characteristics of SRI-treated mothers with (*n* = 23) and without (*n* = 26) plasma drug concentration levels obtained for at least 3 of 5 time-points.

|  | **Drug Levels Analyzed**  **(*n* = 23)** | **Drug Levels Not Analyzed/Obtained**  **(*n* = 26)** | **Test Statistic**  **(p-value)** |
| --- | --- | --- | --- |
| Group (S-D / S-ND) | 16 / 7 | 15 / 11 | *χ^2^*_(1)_ = 0.32 (0.6) |
| Maternal Age (years) | 33.6 ± 5.4 | 35.0 ± 5.8 | *t*_(47)_ = 0.89 (0.4) |
| Maternal Weight at 36-weeks’ (kg) | 86.8 ± 15.0 | 77.0 ± 9.4 | *t*_(47)_ = 2.9 (0.006) |
| Parity (*n*) | 0 (0, 1) | 0 (0, 1) | *W*= 276 (0.6) |
| Education (years) | 17.7 ± 4.3 | 17.7 ± 3.1 | *t*_(47)_ = 0.08 (<0.9) |
| Alcohol During Pregnancy  (*n* total drinks) | 1 (0, 3.5) | 0 (0, 4) | *W* = 333 (0.5) |
| Smoking During Pregnancy (*n*) | 1 | 1 | (1.00) |
| HAM-D score (at 36-weeks’) | 11.0 ± 4.8 | 10.2 ± 4.4 | *t*_(47)_ = 0.44 (0.7) |
| Citalopram (*n*, of 14 total) | 5 | 9 | – |
| Escitalopram (*n*, of 7 total) | 0 | 7 | – |
| Fluoxetine (*n*, of 5 total) | 3 | 2 | – |
| Paroxetine (*n*, of 4 total) | 2 | 2 | – |
| Sertraline (*n*, of 6 total) | 2 | 4 | – |
| Venlafaxine (*n*, of 12 total) | 11 | 1 | – |
| Moclobemide (*n*, of 1 total) | 0 | 1 | – |

*Continuous variables reported as mean ± SD if normally distributed, or median (first, third quartile) if skewed. Categorical variables reported as total number (n). Test statistics, degrees of freedom, and associated p-values are reported for between-group differences using the two sample t-test (t), Chi Square test (χ^2^), Fisher’s Exact test, or Wilcoxon rank sum test (W), where appropriate.*

*S-D, SRI-Depressed group; S-ND, SRI-Non-Depressed group.*

*SRI, serotonin reuptake inhibitor; HAM-D, total score from Hamilton Rating Scale for Depression; kg, kilograms.*

**Supplementary Table 3:** Times of maternal blood collection and the corresponding plasma drug levels (ng/mL) for SRI-treated women (*n* = 23) during the present study at 36-weeks’ gestation.

| **Subject** | **Antidepressant** | **Dose (mg/day)** | **Baseline** | | **Post-Dose 1** | | **Post-Dose 2** | | **Post-Dose 3** | | **Post-Dose 4** | |
| --- | --- | --- | --- | --- | --- | --- | --- | --- | --- | --- | --- | --- |
|  |  |  | Time | Plasma Level | Time | Plasma Level | Time | Plasma Level | Time | Plasma Level | Time | Plasma Level |
| 1 | Citalopram | 40 | 08h08 | 23.02 | 10h47 | 26.05 | 13h25 | 59.51 | – | NA | – | NA |
| 2 | Citalopram | 40 | 08h10 | 54.61 | 10h35 | 53.76 | 13h02 | 342.38 | 13h24 | 284.82 | 15h11 | 95.86 |
| 3 | Citalopram | 60 | 08h04 | 93.50 | 10h21 | 95.82 | 12h48 | 562.45 | 13h06 | 390.12 | 15h09 | 197.89 |
| 4 | Citalopram | 50 | 08h10 | 69.56 | 10h14 | 68.54 | 12h37 | 316.26 | 13h11 | 243.02 | 14h54 | 154.41 |
| 5 | Citalopram | 40 | 07h57 | 53.05 | 10h35 | 49.54 | 12h55 | 90.20 | 13h52 | 89.46 | 14h57 | 83.55 |
| 6 | Fluoxetine | 80 | 07h53 | 424.55 | 10h04 | 531.33 | 12h45 | 1209.88 | 14h45 | 1161.80 | 15h45 | 1280.01 |
| 7 | Fluoxetine | 60 | 07h58 | 306.29 | 09h54 | 320.84 | 12h28 | 406.64 | 13h37 | 359.64 | 14h55 | 643.30 |
| 8 | Fluoxetine | 20 | 07h48 | 95.81 | 09h26 | 88.65 | 11h57 | 119.52 | 12h52 | 152.43 | 14h11 | 115.95 |
| 9 | Sertraline | 200 | 08h25 | 54.78 | 10h43 | 49.07 | 13h13 | 194.21 | 13h55 | 302.19 | 15h22 | 234.77 |
| 10 | Sertraline | 125 | 07h52 | 47.34 | 10h50 | 50.09 | 13h00 | 91.44 | 14h08 | 70.53 | 15h20 | 67.75 |
| 11 | Paroxetine | 30 | 08h54 | 1.73 | 11h08 | 3.58 | 13h27 | 18.92 | 13h50 | 11.95 | 15h38 | 12.61 |
| 12 | Paroxetine | 20 | 07h44 | 27.11 | 9h59 | 25.87 | 12h22 | 29.24 | 13h30 | 25.46 | 14h55 | 24.15 |
| 13 | Venlafaxine | 75 | 08h25 | 156.08 | 10h10 | 94.97 | 13h24 | 172.48 | 13h50 | 167.93 | 15h10 | 168.83 |
| 14 | Venlafaxine | 75 | 08h05 | NA | 10h21 | 0.39 | 12h50 | 15.87 | 13h17 | 14.71 | 14h50 | 13.48 |
| 15 | Venlafaxine | 150 | 08h38 | 4.41 | 10h10 | 3.36 | 12h39 | 32.58 | 13h02 | 41.36 | 14h05 | 40.84 |
| 16 | Venlafaxine | 150 | 08h02 | 11.28 | 10h05 | 31.67 | 12h38 | 75.32 | 13h25 | 63.09 | 14h25 | 54.26 |
| 17 | Venlafaxine | 187.5 | 08h05 | 14.87 | 10h19 | 11.44 | 12h50 | 42.72 | 14h00 | 51.42 | 15h10 | 58.13 |
| 18 | Venlafaxine | 150 | 08h20 | 10.48 | 10h30 | 8.97 | 13h05 | 51.74 | 13h38 | 66.22 | 15h08 | 49.65 |
| 19 | Venlafaxine | 100 | 08h06 | 16.82 | 10h13 | 13.01 | 12h39 | 49.03 | 13h30 | 56.84 | 14h38 | 52.49 |
| 20 | Venlafaxine | 262.5 | 08h10 | 20.49 | 10h31 | 16.58 | 12h02 | 94.99 | 12h56 | 238.14 | 14h05 | 401.53 |
| 21 | Venlafaxine | 150 | 08h05 | 2.31 | – | NA | 13h07 | 26.91 | 13h40 | 26.61 | 14h57 | 28.91 |
| 22 | Venlafaxine | 225 | 07h58 | 472.30 | 10h11 | 483.37 | 12h30 | 696.58 | 13h42 | 829.76 | – | NA |
| 23 | Venlafaxine | 150 | 08h28 | 2.09 | 11h09 | 1.64 | 13h28 | 24.65 | 14h47 | 34.10 | 15h40 | 45.40 |

*Drug levels obtained from high performance liquid chromatography tandem mass spectrometry.*

*NA, Not Available.*
